# Contributing to the debate on categorising shared sanitation facilities as ‘unimproved’: An account based on field researchers’ observations and householders’ opinions in three regions, Tanzania

**DOI:** 10.1371/journal.pone.0185875

**Published:** 2017-11-06

**Authors:** Khalid Massa, Fadhili Kilamile, Emmanuela Safari, Amour Seleman, Anyitike Mwakitalima, Jonas G. Balengayabo, Telemu Kassile, Peter E. Mangesho, Godfrey M. Mubyazi

**Affiliations:** 1 Ministry of Health, Community Development, Gender, Elderly and Children, Dar es Salaam, Tanzania; 2 Ardhi University, School of Environmental Science and Technology, Dar es Salaam, Tanzania; 3 Sokoine University of Agriculture, Faculty of Science, Morogoro, Tanzania; 4 Amani Medical Research Centre, Muheza, Tanzania; 5 National Institute for Medical Research, Department of Health Systems and Policy Research, Dar es Salaam, Tanzania; Weill Cornell Medical College in Qatar, QATAR

## Abstract

**Background:**

Health risks associated with poor sanitation behaviours continue to be reported mostly from low-income countries (LICs). Reports show that various factors limit many people from accessing and using improved latrines, forcing some to opt for sharing latrines with neighbours, others practicing open defecation. Meanwhile, debate prevails on whether shared latrines should be categorised as unimproved according to WHO/UNICEF-JMP criteria. We contribute to this debate based on results from a study undertaken in three regions, Tanzania.

**Materials and methods:**

Data were collected through observations in 1,751 households with latrines, coupled with collection of opinions from heads of such households regarding the latrine-sharing practices. Bivariate and multivariate logistic regression analyses were performed to assess associations between the outcome and possible predictor variables.

**Results:**

Of all 1,751 latrines, 14.6% were shared. Among the shared latrines, 74.2% were found being generally clean as compared to 69.2% of the non-shared ones. Comparing the shared and non-shared latrines, the non-shared latrines were significantly less likely to be found with floors built with permanent materials (OR = 0.73, 95% CI: 0.55, 0.98); washable floors (OR = 0.69; 95% CI: 0.51, 0.93); and lockable doors (OR = 0.73; 95% CI: 0.56, 0.95). Shared latrines were less likely to have floors with faecal matter, functional handwashing facilities (HWFs), HWFs with running water, and roofs; albeit the differences in all these scenarios were not statistically significant. Respondents expressed desire for improved latrines, but also did not find it wrong to share latrines if cleanliness was maintained.

**Conclusion:**

Having an ‘improved’ latrine remains important as JMP recommends, but based on our study findings, we argue that possessing a non-shared latrine neither guarantees safety to its users nor its categorisation as ‘improved’. Instead, the state of the latrine, the construction technology used and the behaviours of the users may be more important.

## Background

### Introduction

The global Sustainable Development Goals (SDGs) which were set around the end of 2015 have specified the need for low and middle-income countries (LMICs) to ensure that an increased proportion of the populations have access to improved sanitation facilities. This is a crucial call as reports from these countries still indicate a high prevalence of people suffering from and dying of infectious diseases. These include diarrhoea, typhoid, schistosomiasis, cholera and intestinal helminthes, all of which are related to poor sanitation and hygiene behaviours at both household and public levels, especially where people share latrines [1,2]. The World Health Organization (WHO) and the United Nations Children’s Fund (UNICEF) through the Joint Monitoring Programme (JMP), define ‘*improved sanitation*’ as a ‘sewer system, septic tank or pit latrine, ventilated improved pit latrine (VIP), composting latrine or pit latrine with a slab that is not shared with other households’. Any latrine, whether a pit without a slab, a platform, a hanging, or a bucket latrine is considered as unimproved [3]. According to JMP criteria, any type of improved sanitation facility is categorised as ‘unimproved’ if it is shared between two or more households [2,4]. However, the United Nations (UN) views the term ‘improved sanitation’ as having context-specific meanings that may differ from one place or social-cultural setting to another. In general terms, improved sanitation includes the lowest-cost option of securing sustainable access to safe, hygienic and convenient facilities and services for excreta and sullage disposal. These conditions provide the desired environment for people to use the facilities with dignity while at the same time ensuring a clean and healthy living environment both at home and in the users’ neighbourhoods [5,6].

The JMP strongly recommends that each household should possess and improved type of latrines, meaning that the latrine should be in acceptable standard of construction, equipped with basic facilities and used only by one household. Any latrine which is shared by two or more households should not be categorized as ‘improved’. This is because such a latrine is more likely to be fouled by some of its users with their excreta. Once this happens, other people who would come to use the facility would be exposed to the risk of infectious diseases such as diarrhoea, typhoid, urinary tract infections (UTI), and several others [7]. In order to avoid sharing a latrine, some people find it better to defecate in open spaces such as bushy areas or along the streets. In so doing, these people increase the chances of spreading the pathogens which are responsible for the transmission of infectious disease to the entire community [8,9].

The JMP’s definition of improved sanitation facility has triggered a debate on what should actually be a more appropriate meaning of improved sanitation. Critics of the JMP’s definition of ‘improved’ sanitation facilities see such a definition as being either narrow or missing some context-specific representations. Such critics (hereafter referred to as experts) include environmental health researchers, academics, environmental health practitioners, public health analysts, and sanitation and hygiene programme officers, just to mention some. These consider ‘an improved sanitation facility’ as one which meets the necessary or basic construction and hygiene conditions that allows the user access and use it conveniently and then leave it clean and undamaged. The convenience and comfort nature of the latrine is referred to here to imply that the latrine concerned should be easily reached, safe and easy to use meanwhile guaranteeing users with protection from possible physical harm or injury that may be caused by either the way it has been constructed (e.g. its physical structure) or an attack from other person(s) who might be venturing in the neighbourhood. It is further suggested that users must be protected from direct contact with human excreta, the latrine should be accessed in a convenient way when needed, and it should be easy to clean and maintain it in case of any damages [6,10].

Globally, about 2.5 billion people (one third of the world population) do not have access to any type of improved sanitation [7,11]. Of these, 732 million are still using latrines that do not meet the basic sanitation and hygiene standards [8]. Open defecation has become a common practice of many people with no access to improved sanitation facilities. Records indicate that around one billion people in the world regularly practice open defecation partly due to either lack of latrines or having difficulties in accessing improved types of latrines [9]. Lack of latrines also forces a number of people from some households to rely on latrines owned by their neighbours. Generally, records from low-income countries (LICs) show that about 16% of the population shares latrines. In the sub-Saharan Africa (SSA) region alone, the proportion of the households sharing latrines is reported to account for 19% of the households sharing latrines in the world [8]. This is one reason that prompts WHO/UNICEF through JMP to urgently require member countries to make sanitation and hygiene issues a priority in their national health policies and programmes. This is intended to increase the number of households with access to improved sanitation [3,12]. Many LICs have positively responded to his requirement. Efforts have been (and continue to be) made to establish policies that aim at improving environmental sanitation and hygiene as previously agreed and in line with the Millennium Development Goal (MDG)– 7c [9]. Some of the efforts which have been made so far, include the provision of public education and sensitization services on issues relating to environmental sanitation and hygiene. This has been done hand in hand with the creation or review of the existing policies and regulations aimed at directing community members to appropriately use improved sanitation facilities and keep the general environment clean. However in practice, there has been a low response of community members to adopt improved sanitation technologies in LICs due to a number of constraining factors. These include poverty, which hinders a number of households from constructing latrines, and preference to open defection which is influenced by social-cultural and traditional norms. Other most commonly or frequently reported constraints include low government prioritization of budgets for, and actual investment in, sanitation management aspects and inadequate enforcement of the existing laws and regulations [1,13].

### Overview on contemporary international debate on the meaning of unimproved latrines

As stated in the introductory section, experts appeal for a review of the WHO/UNICEF JMP’s definition of improved sanitation facilities. The experts are questioning the rationale of categorising every shared latrine as ‘unimproved’ and unsuitable for use [6,8]. In their view, it is impossible to avoid sharing of sanitation facilities in LICs, especially in urban or peri-urban settings as these are areas which are normally congested with people. For example, latrines in public places are usually visited by high number of users such as travellers and businessmen; urban slums and areas located in un-surveyed places host people with diverse social-cultural backgrounds, beliefs and lifestyles living in congested houses. Therefore, sharing a latrine among two or more households for residents in these areas is neither surprising nor avoidable. Instead, the practice is considered a symbol of social solidarity as people see themselves as valuing and respecting each other through communal sharing of facilities including latrines. Others consider the practice of sharing a latrine as a convenient relief from the burden of cleaning the latrine every day since members from different households would now agree to share the responsibility of cleaning the facility through rotation basis [2, 6, 8, 14–16]. Other critics argue that the conventional definition or categorisation of improved latrines may not apply to some local contexts due to technological limitations. Therefore, categorising all latrines found in such contexts as unimproved simply because not all ‘improved’ criteria are met, actually underestimates the efforts made by program authorities and individuals working to improve the conditions and use of latrines previously seen as being poor for use [13]. Another criticism against the JMP’s definition of ‘improved sanitation’ is related to the criterion of ‘the latrine not being shared’, which means that such latrine should be used privately by only one household. As argued earlier, there are situations where members of the same household may not be comfortable with the behaviour of sharing a latrine if they perceive sharing as a taboo; for instance, males sharing a latrine with females, parents or adults sharing a latrine with children, mothers or fathers doing so with daughters or sons in law, and the like [16,17]. On the other hand, other people may perceive sharing latrines as beneficial in maintaining users’ social dignity and at the same time reducing or preventing the health risks associated with open defecation practices [18,19].

Evidence shows that people residing in different socioecological contexts sometimes express different preferences to latrine technology, even for the latrines categorised as ‘improved’ as per JMP’s definition. In Tanzania for example, residents in several communities dislike pit latrines because they find them smelling badly and therefore, uncomfortable to use. They also find the size of the drop-hole to be too small for the user to drop their excreta conveniently. In other contexts, pit-latrines were found not to be liked because of the costs of their construction or other limitations [13].

### Stakeholders’ call for further research in relation to shared sanitation facilities

The continuing debate on what should be regarded as the best definition of ‘improved latrines’ prompted different scholars from public health, environmental sanitation to those with hygiene expertise to suggest for more research to be carried out in different contexts of LMICs in order to come up with local interpretations of the term ‘improved sanitation facility’. Such research may include studies looking at issues such as community perceptions of sanitation and hygiene, types of sanitation facilities they actually use, acceptability of different types of latrines and reasons for use, and their interpretation of improved sanitation facility [6]. More robust and methodologically sound studies are envisaged to come up with additional evidence which is needed to enhance the common understanding of acceptable and critical issues on sanitation and hygiene. The research findings may lead to the establishment of a better or revised definition of ‘improved sanitation facility’ which would take into account specific conditions and contexts of local communities such as cultures, availability or access to resources, perceptions and attitudes towards ‘improved sanitation facility’. Once a commonly acceptable definition is obtained and agreed upon by all stakeholders, it would be possible for policymakers and relevant programme management authorities to identify interventions which would be needed to more appropriately overcome the challenges on matters related to environmental sanitation and hygiene. WHO and UNICEF are among the agencies who advocate for further studies that would employ more scientifically acceptable research methodologies using harmonized questions and research designs to be carried out on this dimension. The authors believe that scientific studies may help them to come up with a reasonable cut-off point within which the aspect of ‘sharing’ a sanitation facility can be seen as hygienically acceptable [3, 6,10]. Kabange and Nkasah [14] argue further that carefully designed and implemented research, can achieve a redefinition of what should realistically be regarded as ‘improved latrine’ and thereby help to correct or advance the currently criticised JMP definition, enhance the national records on improved sanitation coverage and extend acknowledgements of the good work done so far by countries and agencies in the water, sanitation, and hygiene (WASH) field.

Taking into account the above cited experts’ experience and recommendations, we designed and conducted a study in three communities in Tanzania, looking at aspects of latrine conditions, household latrine use behaviours, and household perceptions of the latrine sharing practices. Tanzania is one of the poor countries in SSA where the Ministry of Health (MoH) has maintained the adoption of the JMP definition in their efforts to design policies and ensure implementation of programmes aimed at enhancing household use of improved sanitation technologies. As reported from other LMICs, Tanzania is not free from some traditional and cultural values including norms and taboos (e.g., males sharing a latrine with females, parents or adults sharing a latrine with children, mothers or fathers doing so with daughters or sons in law, etc.) which partly inhibit the acceptability and adaptability of the recommended sanitation technologies [13]. Specifically, our objectives were to interview household members on issues related to sanitation facility sharing practices, and conduct observations on the types and hygiene conditions of the latrines and attached facilities as used by the respective household members in rural and urban settings within three regions. We intended to compare and contrast the present study findings with the findings of the studies conducted elsewhere in Tanzania and abroad and then provide our verdict on the issue of whether or not shared latrines are all unimproved.

## Materials and methods

### Study design and areas

The present study was cross-sectional in design as it did not involve any follow up of events in the study households/communities. It was conducted in the month of July 2014, covering households selected from rural and urban settings in three regions, namely Pwani, Morogoro, and Tanga in Tanzania. Pwani and Morogoro regions are situated in the Eastern zone of the country, and Tanga is in the northern zone. The urban areas covered include the Tanga City Council (CC) in Tanga Region, Kibaha Town Council (TC) in Pwani Region, and Morogoro Municipal Council (MC) in Morogoro Region. Rural areas covered were Bagamoyo District Council (DC) in Pwani, Morogoro DC in Morogoro, Lushoto DC in Tanga. The inclusion of a mixture of rural and urban councils was based on the evidence that there have generally been varying trends and rates of households adopting improved types of sanitation facilities between these settings in Tanzania, with rural settings lagging behind of urban settings [19].

### Study population and sampling strategies

The reporting unit for the study was a household in the sampled regions. However, for a household to be included in the study, it had to have a latrine of any form (regardless of whether it was improved or unimproved). To arrive at this, we produced a list of all households which were reported to have latrines by referring to the National Sanitation Campaign Registers. These registers contain the names of all heads of households for each region in the country.

The selection of the specific regions for the study from each zone was based on a simple random sampling strategy. First, a list of names of all regions in each zone was produced. Then, the name of each region was written on a separate small piece of paper that was put in a separate box. The box with the papers was then shaken thoroughly to allow the papers mix up together ready for random selection. Thereafter, the researchers requested someone who was not part of the research team to pick one piece of paper from the box at random and by so doing, the name of the region on the piece of paper which was picked became the selected region. Following the same procedure, we identified the names of the DCs from each region. Urban councils had to be sampled conveniently as they were the only major town centres and were the capital in each region or district.

A multistage systematic random sampling strategy was used to obtain the number of households in the study, bringing a total of 1,751 households. However, the number of the households sharing latrines as opposed to households which were not sharing latrines at the time of study was not calculated or determined in advance. This was left to be reported from the study as it was one of the specific study objectives. Where the head of the sampled household was absent, a representative member from household and who could respond to our questions was recruited to take part in the study. These heads (or representative heads) of households were selected without researchers knowing in advance socio-demographic characteristics of the respective members such as age, sex, literacy/level of education, occupations or employment status, income, ethnicity, and other backgrounds, for example religion and other cultural values.

To account for how we used a multistage random sampling strategy to arrive at the above specified number of households, we visited the households after following the chain used to identify the study regions and then the study councils. We ensured that in each region, we identified one urban council (a town or municipal council) and one rural council. Then we identified the wards and in each ward the villages and in each village the households. We followed the procedures as the ones used by other researchers to select the study villages and households for their respective study [20].

We estimated the number of households for interview in each region using the formula given by Cochran [21], which is defined as in Eq (1)

$$n=\frac{z^{2}\pi (1-\pi )}{d^{2}}f$$ (1)

where *n* is sample size, *π* is proportion of households with access to a sanitation facility, *f* is design effect, *d* is margin of error, and *z* is standard normal deviate set at 1.96 for 5% level of significance. Ten percent of the calculated sample size was added to cater for the possible non-response rate, leading to a total sample size of 589 households per region. The latter sample had to be redistributed in order to ensure fairness by taking into account the rural-urban differences in the population sizes as available records indicated that the population in rural areas in Tanzania accounted for 70% while in urban areas it accounted for 30% [22]. This resulted in a final total sample of 177 and 412 households for urban and rural settings, respectively. A similar sample determination was used by previous researchers [22,23].

### Data collection methods

#### Recruitment and training of data collection assistants

The data collection process was conducted by the team of five investigators and nine field assistants. The educational/training backgrounds of these assistants were mixed. The team included some members with knowledge in environmental health sciences and others with knowledge in social sciences, all of them had field research experience and demonstrable good interpersonal communication skills. Mastery of Kiswahili -the national language- was considered essential since data collection was planned to be conducted in Kiswahili language. A three-day training session was organised, involving classroom hours and fieldwork to orient and familiarize the field assistants on ethical aspects and other possible field data gathering process, challenges, and outcomes.

#### Data collection procedures and type of data collected

We conducted transient walks/visits to the selected households with a view of observing the presence of latrines as well as the types and conditions of use including hygiene environments of such latrines. This approach was used by other researchers [13, 9]. Our observational visits to selected household latrines went hand in hand with seeking opinions from members of the respective households on issues that were being observed. Household members were given the opportunity to comment on: (i) whether or not the observed latrines were shared with the neighbours; (ii) the suitability for use including safety; (iii) convenience of use such as location of the latrine and; (iv) privacy, comfort, and other qualities while using the latrine. The latter include building materials, other structural aspects surrounding the latrine including the presence of human excreta, convenience of cleaning the latrine and maintaining it in case of damage, distance of the latrine from the main (residential) house, and degree of member’s satisfaction with each of these conditions. As done by other researchers, the informants in all these aspects were also asked for their opinion as to whether their latrine was ‘improved’ or ‘unimproved’ when interpreted according to their own local contextual environment [24]. Thus, personal (unstructured and informal) communications (abbreviated as per comm.) with the respective household members were conducted in an attempt to seek additional opinions from the respondents that could corroborate the data which were obtained from direct observations of the latrine. The relevance of using per comm. was justified by the experience gained from the pre-testing and pilot survey stages of the present study whereby the respondents were found to be more honest in expressing their thoughts while accounting for issues relating to sanitation and hygiene behaviors. They could not report in the same way when responding to questionnaire-based face-to-face interviews. This is partly because most of the questions were closed and thus, did not give scope for the respondents to give detailed answers that seemed to them appropriate.

The observation process was guided by an observational checklist, and as done in other studies, we followed the WHO/UNICEF JMP criteria for assessing such aspects as mode of construction or type of the latrine [9,16]. The types of latrines, which were observed, were traditional pit, improved pit, ventilated improved pit (VIP), water closet or ecological sanitation type or others. Hygiene status included the presence or absence of handwashing facilities (HWFs); presence of running water and soap were also observed. Other observed aspects included whether or not the door could be locked from the inside, whether or not the door could allow a person from outside to see through, whether or not there was a room, and the presence of human excreta or any other unhygienic disposable substances [3]. HWFs were observed to determine whether or not they were functioning at the time of this study. Functionality was assessed in terms of whether or not the facility was in good usable state whenever the need arose and whether there was water supply and evidence of being in use. To countercheck in which condition/state each of these features was during the period prior to this study, the respondents from our host households were consulted.

The latrine cleanliness condition was assessed based on several factors. For example, attention was paid on whether or not there was a clean slab and at the same time there was no faecal matter or traces of urine on that slab.

Latrine accessibility was evaluated by assessing the presence or absence of any obstacles that seemed to cause difficulties or inconveniences for the members of the respective households to visit or use the facility. The researchers were interested in observing whether the respective latrine was located within a 10 meters radius from the main (residential) house.

Other aspects or conditions observed include suitability of the latrine for use by disabled people. Furthermore, features relating to the superstructure of the facility including its physical appearances in terms of construction design and the materials used to build it were assessed by taking notes of the presence or absence of cracks on the walls, reliable roof, and similar aspects.

Answers to each of the above mentioned conditions as per the checklist used in the assessment for this study was assigned two codes, namely, ‘Yes’ if the stated element was present or ‘No’ if it was lacking.

Before their use in the actual observational survey, the research instruments were pre-tested at two stages. First, the instruments were administered to staff working for the Ministry of Health, Community Development, Gender, Elderly, and Children (MoHCDGEC). These members were asked in advance as to whether they were heads of their respective households, and after confirmation, they were eligible for the pre-testing. Among these were those who were living as tenants in rented houses and reported to have been sharing latrines with other tenants living in the apartments of the same building or neighbours from the neighbouring houses. Second after pre-testing, the revised tools were tried out again in a field survey. The survey was conducted in Mtimbwani sub-village, a hamlet located in one of rural areas of Mkinga District in Tanga Region and in Nguvumali neighbourhood located in Tanga City.

### Data management—Handling, cleaning and analysis processes

Data entry was performed on participatory basis involving each data collector through the open data kit (ODK) software version 2.0 that was installed on smartphones to facilitate the collection and transferring of data into the database that was hosted at the National Institute for Medical Research, Amani Center. Thereafter, data were exported to Epi Info^™^ version 7.1.4.0 (Centres for Disease Control and Prevention, Atlanta USA) for cleaning before analysis. Responses to questionnaire-based open-ended questions were later coded to allow easy entry in the computer for quantitative analysis to obtain the frequencies of some of the answers of interest. However, statements which seemed to be important for presentation in the report either in the form of quotations or other qualitative narrations were recorded by hand in a separate notebook.

The analysis of the coded data from both the observational checklist and the questionnaire was also facilitated by the Epi Info statistical software. Qualitative data such as those obtained through personal communications were processed manually by applying a qualitative content analysis approach [25]. Descriptive statistics were used to describe the characteristics of the data. In particular, frequencies and percentages were calculated. Chi-square tests were also done to assess whether or not there were statistically significant differences in the results obtained from the cross-tabulated variables (sanitation sharing status and characteristics of latrines). Thereafter, binary and multivariate logistic regression analyses (LRA) were carried out to trace the possible influence of specific independent variables over certain dependent/ outcome variable. The multivariate regression analyses were conducted adjusting for the same variables used in the binary regression analysis. Unadjusted and adjusted odds ratios (ORs) and 95% confidence intervals (CIs) were estimated from the binary and multivariate logistic regression analyses. The outcome variable of interest in this study was whether the latrine observed was being ‘shared’ or was used ‘privately’ (i.e. unshared)’. This was a binary outcome in nature as it was assigned codes ‘Yes’ and ‘No’. Similar codes were used when assessing whether or not the observed latrine had the following conditions: functioning HWFs, faecal contamination on the floor, lockable doors from inside, washable floor, human faeces on the surface, and soap for washing hands after using the latrine; and whether or not the latrine had any of the rest of the elements/conditions as stated along with the data in Table 1. The independent variables registered include such elements as the respondent’s age, sex, occupation/employment, and education, place of residence (i.e. rural or urban), and other demographic characteristics. Qualitative data from open-ended questions on the questionnaire and personal communications with the members of the households were analysed manually, and as mentioned earlier, this was achieved by looking at and interpreting the content of the text data as per the summative qualitative content analysis approach [25]. With the latter approach, textual data were examined by counting some commonly or similarly mentioned elements/conditions with the aim of comparing the views expressed by the rural and urban respondents, male versus female respondents, and adults versus young people (aged 15–24 years). Useful quotations were identified and presented selectively to reflect the general viewpoints of the individual respondents on a particular aspect under study.

**Table 1:** Comparison of characteristics between shared and non-shared sanitation facilities as observed in three regions.

Characteristics	Sanitation facility		χ^2^ (p)-value	OR (95% CI)
	Shared n (%)	Non-Shared n (%)		
**Wall construction material**				
Not permanent	77(30.1%)	502(33.6%)		1
Permanent	179(69.9%)	993(66.4%)	1.21(0.27)	0.85(0.64, 1.13)
**Floor material**				
Not permanent	71(27.7%)	514(34.4%)		1
Permanent	185(72.3%)	981(65.6%)	4.3(0.04)	0.73(0.55, 0.98)
**Presence of a lockable door**				
Absent	106(41.4%)	735(49.2%)		1
Present	150(58.6%)	760(50.8%)	5.26(0.02)	0.73(0.56, 0.95)
**Presence of a roof**				
Absent	106(41.4%)	594(39.7%)		1
Present	150(58.6%)	901(60.3%)	0.25(0.61)	1.07(0.82, 1.40)
**Hand washing facility**				
Absent	245(95.7%)	1395 (93.3%)		1
Present	11(4.3%)	100(6.7%)	2.11(0.15)	1.60(0.84, 3.02)
**Functional HWF**				
Not functional	4(36.4%)	21(21.0%)		1
Functional	7(63.6%)	79(79.0%)	1.34(0.25)	2.15(0.57, 8.04)
**Position of the HWF**				
Not outside the toilet	2(18.2%)	14(14.0%)		1
Outside the toilet	9(81.8%)	86(86.0%)	0.14(0.71)	1.36(0.27, 6.99)
**HWF with running water**				
No running water	4(36.4%)	32(32.0%)		1
Running water available	7(63.6%)	68(68.0%)	0.44(0.5)	1.21(0.33, 4.45)
**Presence of soap**				
Not present	6(54.5%)	50(50.0%)		1
Present	5(45.4%)	50(50.0%)	0.08(0.77)	1.20(0.34, 4.19)
**Human faeces on surfaces**				
Not present	242(94.5%)	1397 (93.4%)		1
Present	14 (5.5%)	98 (6.6%)	0.43(0.51)	1.21(0.68, 2.16)
**Human faeces outside**				
Faeces not seen	250(97.7)	1468(98.2)		1
Faeces seen	6 (2.3)	27(1.8)	0.34(0.55)	0.77(0.31, 1.87)
**Washable floor**				
Not washable	68(26.6%)	513(34.3%)		1
Washable	188(73.4%)	982(65.7%)	5.92 (0.01)	0.69(0.51, 0.93)
**Cleanliness status**				
Dirty	66(25.8%)	461(30.8%)		1
Clean	190(74.2%)	1034(69.2%)	2.6(0.1)	0.78(0.58, 1.05)
**Accessibility**				
Not accessible	41(16.0%)	137(9.2%)		1
Accessible	215(83.9%)	1358(90.8%)	11.2(0.000)	1.89(1.29, 2.75)
**Usability of toilet to people with disability**				
Not user friendly	157(61.3%)	1006(67.3%)		1
User friendly	99(38.7%)	489(32.7%)	3.48(0.06)	0.77(0.59, 1.01)
**Used all the times**				
No	68(26.6%)	380(25.4%)		1
Yes	188(73.4%)	1115(74.6%)	0.15(0.69)	1.06(0.78, 1.43)

Due to their immense nature, the data obtained through questionnaire-based interviews with heads or representative heads of households were presented in a separate manuscript. The present paper shows and discusses data from field observations which were carried out by the researchers on the types of the latrines used at household level in the urban and rural communities. The key elements observed are summarized in Table 1. These are discussed in combination with qualitative data based on the opinions given by members of the households visited with whom informal interviews (personal communications) were conducted.

### Ethical considerations

The ethical clearance (Ref. No. NIMR/HQ/R.8a/Vol. IX/1538) for this study was obtained from the Medical Research Coordinating Committee (MRCC), under the MoHCDGEC in Tanzania hosted by the National Institute for Medical research (NIMR). Regional and Local Government Authorities were informed in advance about the study and were given copies of the national ethical clearance certificate for this study along with liaising with them for the research team to obtain official permission to implement the study in their respective areas of jurisdiction. Permission was granted by these authorities. At household level, respondents were requested for their consent to participate in the study and their latrine facilities to be visited for observation. All the respondents who freely chose to participate in the study gave verbal informed consent. Oral consent was considered sufficient in this study as no risk to the respondents was expected in the study and because the research did not involve any procedures which would have required written consent. For this reason, participant consent was not recorded. In general, the informed consent process adhered to the stipulated international and national guidelines—including, among other requirements, not to force or unduly influence anybody to participate in the study population; anonymity of the respondents, and confidentially of some information were observed [26].

## Results and discussion

### Latrine design and building materials

Of the 1,751 latrines that were inspected, 256 (14.6%) were found to be shared between two or more households. This was confirmed by the respective members of the households. As for the status of the superstructures of sanitation facilities, about two thirds (n = 1,172; 66.9%) of the total latrines were found to be constructed with permanent materials including cemented blocks, burnt soil-bricks, and iron sheets. There was a slightly larger proportion (n = 179; 69.9%) of the shared latrines than the non-shared (private) ones (n = 993; 66.4%) with walls made of permanent materials. These latrines had floors made of concrete slabs that were finished with cement or tiles. The roofs of these facilities were covered with corrugated iron sheets. The facilities which were either found or reported to be shared were mostly in poor state of construction (Table 1) and posed higher risk to their users. That is why one could disqualify them from being ‘improved’ as per JMP’s criteria. However, we did not conduct a biological assessment to ascertain the magnitude of the risks associated with the use of shared latrines as opposed to non-shared (privately used) ones. This might have helped the experts to confidently attach the risks to households that were sharing and rather than those which were not sharing latrine facilities [7,9,10].

Evidence shows that the type of sanitation facilities may inhibit or motivate household members to use such facilities. Other potential influencing factors include the cultural and psychosocial determinants of sanitation facility use in different local contexts. People may not show preference to even use a latrine that is considered or categorised to be of modern type (an ‘improved’ one) if they find that there is no separation between the rooms for men and women. In certain situations, a pit latrine may not be preferred by the targeted users, especially the women due to cultural limitations such as taboo or religious values limiting them to do so [10,15,17,27]

### Latrine cleanliness and convenience of use

A total of 66 (25.8%) and 461 (30.8%) of the observed shared and the non-shared sanitation facilities, respectively, were found to be clean. A bivariate analysis indicated that the odds of finding the shared sanitation facilities clean were lower as compared to the non-shared ones. However, the observed difference was not statistically significant (OR = 0.78; 95% CI: 0.58, 1.05) (Table 1). Similar results were observed in the multivariate analysis from which the likelihood of shared latrines being found clean, adjusting for other variables, appeared to be lower than that of non-shared latrines (OR = 0.96; 95% CI: 0.63, 1.46) (Table 2).

**Table 2:** Adjusted ORs and 95% CIs of sharing a sanitation facility among households in three regions.

Characteristics	OR (95% CI)	P-value
**Wall construction material**		
Not permanent	1	0.6505
Permanent	1.14 (0.64, 2.1)	
**Floor material**		
Not permanent	1	0.2495
Permanent	0.70 (0.34, 1.3)	
**Presence of a lockable door**		
No	1	0.0248
Yes	0.62 (0.40, 0.94)	
**Presence of a roof**		
No	1	0.0455
Yes	1.6 (1.0, 2.4)	
**Hand washing facility**		
Not present	1	0.1331
Present	1.65 (0.86, 3.2)	
**Human faeces on surfaces**		
Not present	1	0.7824
Present	0.91 (0.48, 1.74)	
**Human faeces outside**		
Not present	1	0.7261
Present	0.84 (0.33, 2.2)	
**Washable floor**		
No	1	0.2142
Yes	0.73 (0.45, 1.20)	
**Cleanliness status**		
Not clean	1	0.8399
Clean	0.96 (0.63, 1.46)	
**Accessibility**		
Not accessible	1	0.0001
Accessible	2.3 (1.48, 3.43)	
**Usability of toilet to people with disability**		
Not user friendly	1	0.0793
User friendly	0.76 (0.56, 1.03)	
**Used all the times**		
No	1	0.5860
Yes	1.12 (0.74, 1.69)	

Human faeces outside the latrine were found in 6 (2.3%) of shared latrines and in 27 (1.8%) of non-shared ones. The likelihood of faecal maters to be found in shared latrines was 0.77 times that of non-shared latrines, although the difference in this case was also not statistically significant from both binary and multivariate regression analysis results: (OR = 0.77; 95 CI: 0.31, 1.87) and (OR = 0.84; 95% CI: 0.32, 2.18), respectively. Inside the facilities, it was found that 14 (5.5%) of the shared latrines had traces of human faeces on the floor. A similar situation to the latter was noted in 98 (6.6%) of non-shared latrines. Logistic regression analysis results from the binary and multivariate models indicated that the likelihood of faecal matter to be found inside the shared latrines was less than was the case inside the non-shared latrines. This was contrary to our a priori expectations based on common sense. Even though the observed difference in the latter case was not statistically significant: (OR = 1.21; 95% CI: 0.68, 2.16) for bivariate and (OR = 0.91; 95% CI: 0.48, 1.74) for multivariate, similar observations were reported from other studies. For example, in one study conducted in recent years in Tanzania, researchers found shared sanitation facilities (latrines) being less contaminated with faecal materials and thus, being less likely to expose users to infections than was the case with the non-shared ones [9]. Similarly, a study undertaken by Tumwebaze and Mosler in Uganda indicated that shared latrines were more likely to be found clean than the non-shared ones [15]. In our study, 188 (73.4%) of the shared latrines had washable floors as opposed to 982 (65.7%) of the non-shared ones, and the difference observed was statistically significant in the bivariate results (OR = 1.44; 95% CI: 1.07, 1.94), but statistically insignificant in the multivariate analysis (OR = 0.73; 95% CI: 0.45, 1.20). Having a washable floor in the latrine of whatever type has been shown to be a requirement for easy or convenient cleaning and may increase the chances for attracting people to use the latrine [15]. However in practice, this may not be always the case since not all well-constructed latrines are necessarily cleaned by the users [9].

Many latrines both shared and non-shared ones were not convenient for use by the people with disabilities. However, non-shared latrines were less likely to be useful for people with disabilities compared to shared latrines. It was noted that only 99 (38.7%) and 489 (32.7%) of shared and non-shared latrines, respectively, were user-friendly to people with disabilities. However, the difference was not statistically significant for both bivariate (OR = 0.77; 95% CI: 0.59, 1.01) and multivariate (OR = 0.77; 95% CI: 0.59, 1.01) analyses.

### Nature of latrine construction materials, safety and accessibility for use

Of the latrines found to have been built with permanent floor materials, 185 (72.3%) were in the shared category while 981 (65.6%) were in the non-shared ones. Bivariate and multivariate regression analysis results showed that shared latrine were 0.73 and 0.70 times respectively more likely to have floors built with permanent materials than the non-shared ones. The difference was statistically not significant in both the bivariate (OR = 0.73; 95% CI: 0.55, 0.98) and the multivariate (OR = 0.70; 95% CI: 0.37, 1.30) analyses. Around two-fifths of the shared latrines (n = 106; 41.4%) and non-shared latrines (n = 594; 39.7%) had no roofs. As for the accessibility, 68 (26.6%) of the shared latrines were not physically accessible to users at all times, and almost the same result was found for the non-shared ones (n = 380; 25.4%). As reported by the respondents, the main hindrance to household members’ from reaching the latrines for use was the location of latrines from the residential houses. The distant of the latrine forced people to visit the neighbours’ latrines. Occasionally, neighbours’ latrines were found to be either locked or in use. The following statements testify:

“*My friend*, *don’t tell me*! *We always pray not to face a diarrhoeal problem; for if this happens while you have no direct access to a latrine because it is either occupied or locked*, *shame can fall upon you*”

(A lady at Kombezi neighbourhood in Makorora ward, Tanga City).

This was generated from a question that was designed to solicit information as to why the respondent did not like sharing a latrine.

“*Do you think those who relieve themselves in open spaces do so willingly*? *Even if it were you*, *what could you do in situations whereby you are so pressured by the urine or feel urgently to go for a long call but finding no latrine around*? *Obviously*, *you can go to a hiding place and quickly deposit the matter for you to get relieved*!”

(A mother aged 30–35 years, Boma neighbourhood in Mazimbu Ward, Morogoro MC).

The research team confirmed through observations the reports given by these respondents regarding the locations of the latrines, the spread of human excreta in some open spaces, lack of lockable doors, and in some latrines doors were found closed and locked. Similar experiences to these conditions as reported from urban centres were reported by members of the households whose latrines were also observed in rural settings. The respondents in the latter category were concerned about safety of using the latrines during odd times such as night hours or rainy times, particularly if the latrine was located at a distance from the living house. It was further testified by a number of respondents in the urban centres that the habit of locking the latrines was sometimes found to be a necessary measure of preventing passers-by from trespassing and using the latrines without taking good care of the latrine after use.

### Privacy

**Fig 1:**
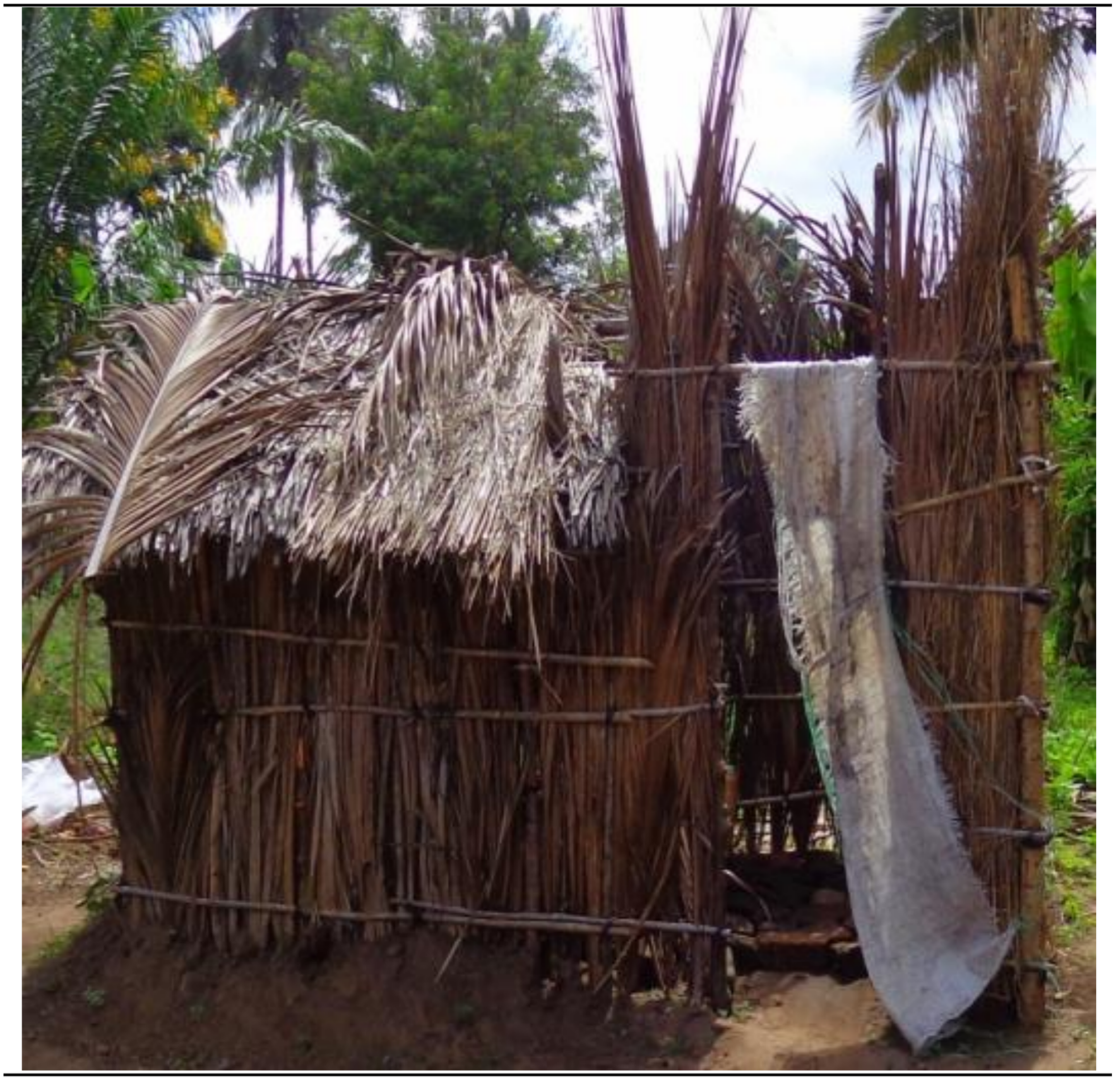
A pit latrine built with temporary materials and lacking door shutter in one of the study areas, Tanzania.

A total of 106 (41.4%) and 735 (49.2%) of the shared and non-shared latrines, respectively, did not have lockable doors. The likelihood of finding latrines without lockable doors and therefore, limiting users’ privacy while using the facility was higher in non-shared latrines than in the shared latrines. The difference was statistically significant in both the bivariate (OR = 0.73; 95% CI: 0.56, 0.95) and multivariate (OR = 0.62; 95% CI: 0.40, 0.94) analyses. No enquiries were done using a questionnaire-based interview approach to establish the reasons for not putting lockable doors in the respective latrines. Nonetheless, opinions obtained through informal communications with several household members revealed that chances for the users of encountering interferences by people from outside, including those living in the neighbouring households or by members of the same household were higher in the shared latrines than was the case in the non-shared latrines. Therefore, in the quest for maintaining privacy, some of the people who could not tolerate using the latrine without lockable doors opted for open spaces in order to relieve themselves. It was also reported that the latrines in multi-habited and latrine sharing compounds, including rented houses accommodating several families had higher chances of being found with lockable doors. In these houses, it is likely for the tenants to complain and force their landlord to fix a lockable door. This is contrary to members living in households without shared latrines as these may not experience the inconvenience of interferences by passers-by while using the facility and therefore, they did not see the urgency of fixing lockable doors. Additional reports as reported by the respondents from the households sharing latrines indicated that their latrines did not have lockable doors- instead a piece of cloth (e.g. khanga, towel or shirts) would be hung at the entrance of the latrine to serve as a signal that the latrine is occupied at the moment and that it is not available to other people who would like to enter and use it. This was confirmed by the investigators during the observation exercise. Furthermore, some of the shared and non-shared latrines which were found without lockable doors were found fitted with the improvised locally available protection materials such as old bed sheets and pieces of sisal mat/sacks at the entrance (Fig 1).

Below, are some of the concerns expressed by the respondents, expressing the feelings they had on the issue of privacy while using the latrine facility:

“*Just imagine if it were you using the latrine you have seen*, *could you tolerate the landlord who collects rent every month but does not value his tenants by not fitting a door*? *I’m telling you—we have cried and cried but no action has been taken*, *leaving endless nuisance to us*!”

(A tenant, Makorora neighbourhood, Tanga City).

“*Anyway*, *that is how we live*, *what do you expect*? *You know living in ‘Uswahilini’ (meaning living in squatter and congested residential places) is a big problem because of lack of privacy both within the houses and in the outside environment*. *You are forced to wait for someone to come out of the latrine for you to go in*, *and at times you may be pushing while defecating and while unprepared someone suddenly pulls up the curtain to move in*, *and by so doing he sees you naked; what a shame*!

(A middle aged lady, Bagamoyo DC).

The above testimonies concur with the findings reported in other studies that were previously conducted in Tanzania. These findings reflect how privacy is valued and how privacy may influence residents of a given home or community to choose either to use or not to use a shared latrine and opt for open space for defecation. As other analysts observe, the concerns about privacy while using a latrine may be higher if other factors are taken into consideration. These include the safety of using the latrine, the perceived health benefits of sharing or not sharing a latrine as well as whether or not it is prestigious (dignifying) to use a private or public latrine [20,13]. For instance, it is documented from studies conducted elsewhere that in attempt to avoid being seen by other people while either on the way to use or to be seen using the facility, a considerable number of people in rural and urban areas decide to refrain from using the latrine. Instead, such people opt for open defecation [16]. On the other hand, lack of privacy in these unlockable doors led to an increased chance for people to opt for open defecation, which in turn led to an increased exposure of such people to the risks of snake bites and or other forms of intimidation. These incidents have both physical and psychological consequences [24].

### Handwashing facilities

**Fig 2:**
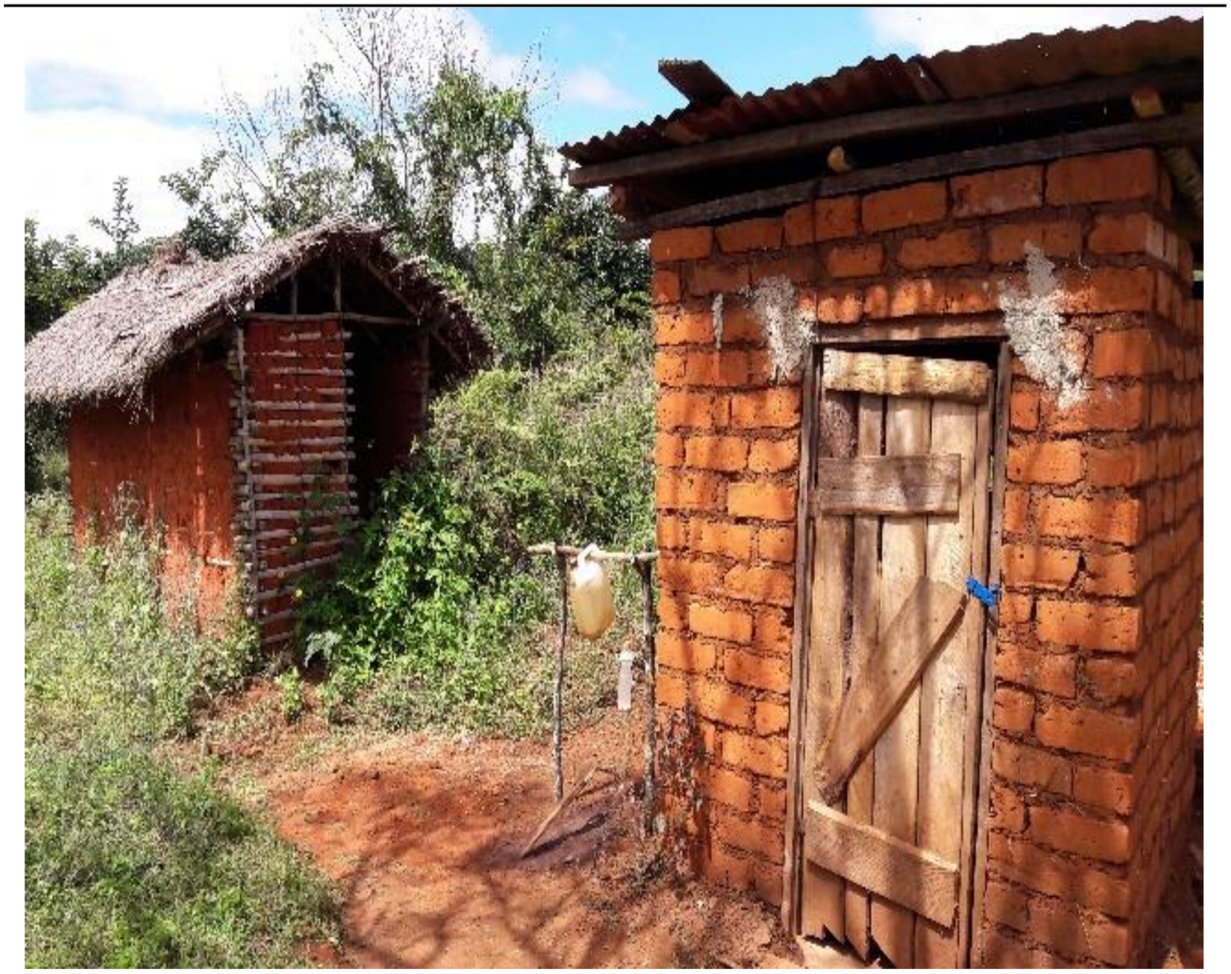
A newly constructed toilet after discarding the old one following the National Sanitation Campaign reported by a household member in Lushoto District, Tanzania.

Overall, the availability of HWFs for users of shared and non-shared latrine was commonly low, accounting for 112 (6.4%) amongst the 1,751 latrine visited. Moreover, 86 (4.9%) of the observed latrines in the study area were found to have functioning HWFs, the majority [79 (91.9%)] of which were found amongst the non-shared latrines. Some of the latrines, which were found with functioning HWFs lacked water in taps, tippy-taps and tanks with a tap (hereafter referred to as ‘running water’). Participants who were asked to comment on the HWF problem appreciated the fact that users of the latrines which lack HWFs could expose users to infection carrying pathogens that might be transmitted to other people. Interestingly, the respondents seemed to recognise that leaving the latrine with unwashed hands could pose a risk to one’s health. To emphasise this point, one respondent showed the researchers a new constructed latrine (Fig 2) after coming to a decision of discarding the old one. The old latrine had no HWFs, a lockable door or improved roof. To the researchers, this sounded very encouraging as it reflected the role played by public health promotion programmes including the National Sanitation Campaign to educate and sensitize the public on sanitation and hygiene.

Furthermore, testimonies were obtained from the respondents regarding the prevailing and very common situations whereby latrine users were being required or forced to wash their hands using the water reserved for anal cleansing. Normally, this water is stored in containers such as buckets which were placed inside the latrine. In the event that the water is emptied in the container after being used, people find themselves being forced to bring their own water from outside. Below, are some of the statements testifying this experience as expressed by some of the respondents:

“*I don’t know what to say about some people*. *They contaminate the water after relieving themselves*. *We are all required to use small vessel to fetch water from the container available for washing our secret places and hands*, *but you find traces of urine or faeces either in the main vessel containing the water for everyone to use or in the small vessel that is normally used for fetching the water from the container either for washing hands or anal cleansing*. *We are surely in trouble*, *and I can’t imagine when people will learn to be civilized*!”

(A gentle lady, Kongowe, Kibaha TC).

**Fig 3:**
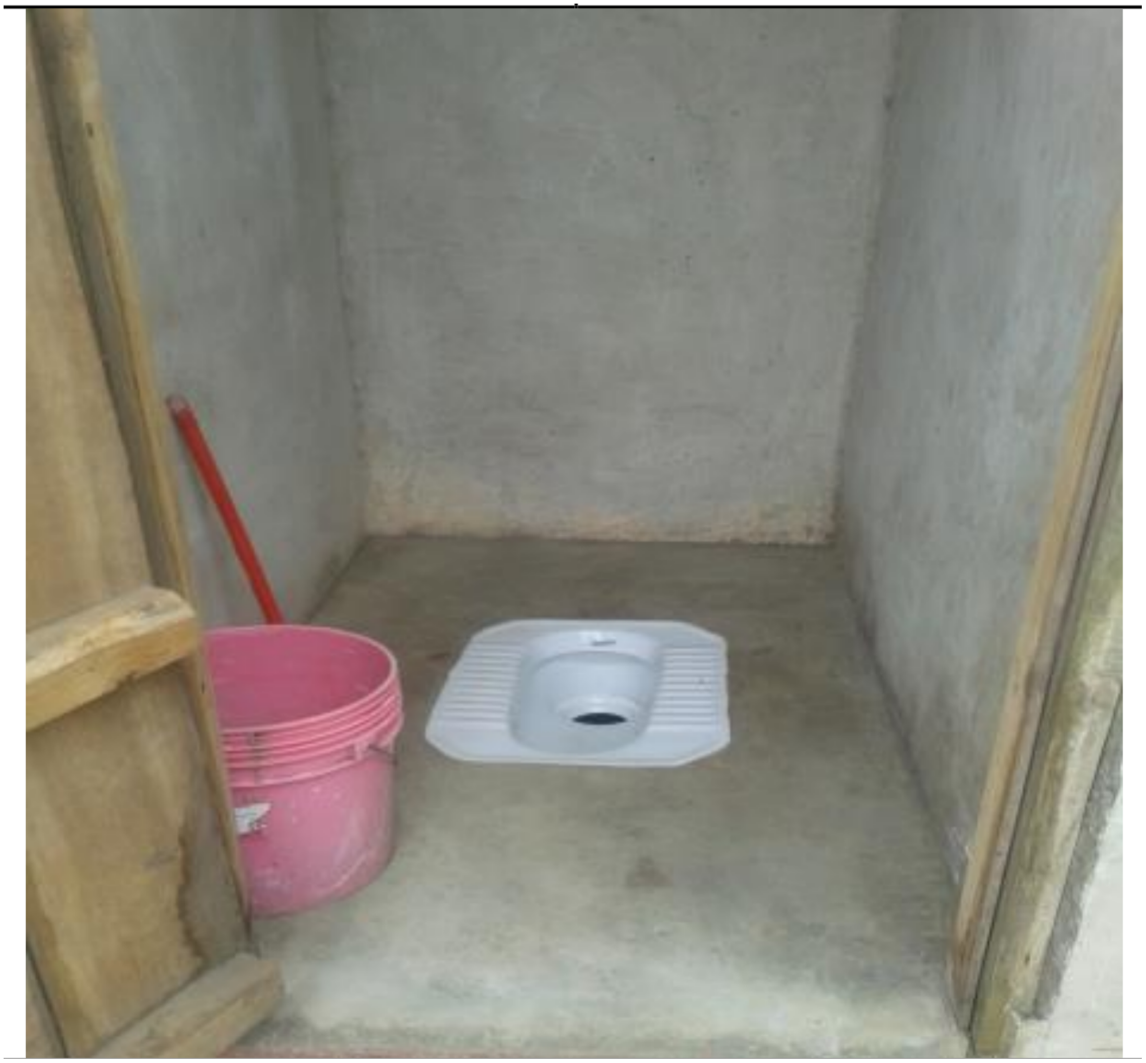
A toilet with a bucket for keeping cleansing water inside but users may take it for granted as an alternative to having a HWF.

As further reported and then confirmed by the findings of the present study that even if the latrine has an attractive building outlook, it may still lack the needed HWFs or reliable water supply system. It is unfortunate that not all users of the respective latrine would dare or find it needful to complain and take necessary actions against this deficiency. Some people did not seem to care about the safety of the water which was available in the main or minor container/vessel, all it matters for them is that there is some water for handwashing after using the latrine (Fig 3).

Handwashing facilities were more likely to be placed outside the latrines, particularly in non-shared as compared to shared latrines. However, the difference was not statistically significant (OR = 1.36; 95% CI: 0.27, 6.99). Lack of soap for handwashing was noted in more than half of the shared types of the latrines observed and in about a half of the non-shared ones. As the results from bivariate LRA indicate, the presence of soap was less likely to be noted in the non-shared latrines as compared to the shared latrines (OR = 1.20; 95% CI: 0.34, 4.19). Although the observed difference in the latter case was not statistically significant, we can still work out the possible reasons for this practice basing on our common sense and experience. For example, people may perceive the risk of using shared latrines to be higher than that of using a non-shared latrine. Such people may see the need for putting soap and other detergents in the shared latrines for hygiene and safety reasons. The practice of using shared latrines may prompt the users of such latrines to remind each other of their latrine cleaning duty on rotation basis following the agreed upon cleaning roster. In this way, it is easy to ensure that persons or households on duty supply the washing materials including soap in the latrine. This may not be the case in non-shared latrines. Due to lack of duty roster for latrine cleaning, the members of the respective households may either forget or may not care to perform their duty. They may do so because of not perceiving the risk resulting from failure to clean the latrine to a high standard [15]. Opinions were obtained from some of the household members who responded to the question as to why soap was sometimes not available in the latrines. As the findings revealed, sometimes pieces of soap were put in the latrine, but in the shared latrines, certain people seemed to misuse these toilet facilities, therefore, discouraging members from other households to supply additional facilities before their time is due as per the roster agreed upon among the latrine sharing households. As reported by the respondents, failure of putting soap in the latrine was not accidental. For example, there are members who were prepared to use the soap economically, and others thought that by supplying soaps in the latrine they were saving resources on their side:

“*You know*, *some latrines like ours as you have seen it are at the same time used as bathrooms*. *So*, *sharing a latrine is a serious conundrum since if you leave a piece of soap for handwashing after defecation*, *some tenants use it for bathing as they want to save cost of buying bathing soap*. *As a result*, *some of us decide to move in and out with our soap after solving our problem at hand*”

(A 45 year gentleman, at Boma neighbourhood, Morogoro MC).

The risks of contracting infections are increased if hands are not washed with soap after using the latrine. This may be confirmed if a microbiological study is conducted. For example, a study conducted recently in Britain established that cases of norovirus, gastroenteritis, MRSA, E.*coli* and swine flu, which occurred in the country were associated with dirty hands. Furthermore, school children were found not accessing soap in the school latrines [24].

As reported in the sister manuscript submitted to BMC Public Health Journal with data from the questionnaire-based interviews, some members of the households perceived that the practice of sharing a latrine could be tolerated provided that the latrine users were civilised to keep it clean after use to allow other people to use it. However, it was observed that the shared latrines were not always cleaned after use. As argued, young children either forget to leave the latrine clean or find it impossible to do so due to lack of water for flushing the excreta. The following experiences were also noted from adult individuals as well. Carelessness was reported to be one of the reasons for the latrines to be left unclean after use by adults who are expected to observe sanitation and hygiene conditions:

“*You may have a latrine used by a single family*, *but still find it unclean if the family members don’t care to strictly observe sanitation and hygiene conditions*. *In contrast*, *you can visit the one shared by several households but find it being very clean because members from the respective households have organized themselves to clean it on rotational basis*”

(A resident who identified himself as a member of the village health committee, Lushoto DC, Tanga).

**“***Do you think that issues of sanitation or hygiene you are investigating on can be forced to the people whose upbringing did not influence them to habitually behave so*? *Haven’t you visited respectable persons but find their latrines being dirty for they do not take trouble to clean it immediately after use*? *I hate it*! *How comes one leaves the latrine without at least flushing the water*? *This could only be tolerated for public latrines*!”

(A male veteran and head of household, Kongowe, Kibaha TC, Pwani).

In summary, ‘an improved type of latrine’ from the viewpoint of the respondents in the present study seems to mean the one that is well constructed, convenient at the time and point of use, and that this can be maintained or renovated. Any construction involving some renovation on the old superstructure was also perceived as being an improved one so long as it is a more user-friendly while using it. These views support the conclusion made previously by the analysts based on the evidence garnered from other LICs. In their review, it was noted that the latrines shared by a small number of households (at least 2 and at most 4) if consistently maintained and cleaned can still satisfy the needs of the users. Such users may feel even more comfortable with shared latrines than using non-shared ones that are not regularly cleaned. This means, context specific considerations are necessary when interpreting the word ‘improved’ or ‘unimproved’ latrine. Therefore, program authorities may need to put more efforts towards better management of the existing types of latrines in terms of maintenance, cleanliness, and other safety measures. This may sound more important or more basic to the poor people living in resource constrained settings of LMICs than imposing rules, bylaws and guidelines strictly restricting them to share latrines with neighbours. After all, this is almost practically impossible in places where people have been and are likely to remain living in social bondages and at the same time being constrained by resource and spaces for constructing non-shared latrines [2, 4,14,15].

### Limitations and strengths of the present study

We attempted to avoid bias in the selection processes by employing a random sampling strategy in the study areas and households. We were also able to demonstrate the results from the experience of rural and urban populations on matters relating to using shared and private latrines at a household level. Despite the interesting findings presented here, the current study has some limitations. The main ones are related to the study design, sampling strategies, data collection techniques, and area of coverage. First of all, the shortage of financial budget for the study made it difficult cover many regions and zones, or a larger number of households within the selected regions. Secondly, the cross-sectional nature of the study could not allow cohort visits to be made to establish whether or not some of the reported behaviours, for example, failure or ability of the household members to clean the latrines on regular basis after use were evident. Thirdly, a meaningful analysis and interpretation of the statistical data seemed to be possible when dealing with data covering the total number of households visited, but not when dealing with small (sub—samples) based on the treatment of the data for each district separately. Covering three regions and two districts only in each region may raise questions about study representation of the entire household population in the country. We have conducted a multivariate regression analysis (MVRA) to supplement the results from the bivariate regression analysis with the aim of strengthening the interpretations made from the data gathered. Fourthly, the study would probably have gained more useful information if it was supplemented by a microbiological investigation to establish the magnitude of the biological risks associated with the practice of using a shared latrine as opposed to the use of non-shared latrines as done in other studies [13,28,29].

## Conclusion and policy implications

This study confirms that sharing of latrines is a common practice in both urban and rural community settings in Tanzania, as reported from studies conducted by other researchers. From these study findings, we argue that whether or not a shared latrine should be regarded as ‘unimproved’ depends on the viewpoint of the people in a given socioeconomic and ecological setting. A wholesale adoption of the JMP’s definition of an ‘improved latrine’ to enforce programmes aimed at encouraging people to avoid sharing latrines is practically impossible in all social-cultural and resource-constrained contexts. People may not find the practice of sharing a latrine an issue, if the facility is well constructed, maintained for cleanliness, and conveniently accessible at the time of need for use. Otherwise, a well-constructed latrine that is used by only one person or only by a single household may still be unused if other conditions are not met. For example, convenience of accessing it physically, cleanness, and other safety conditions need to be available.

However, the respondents to this study expressed concern about the possible risks associated with the use of shared latrines that have no lockable doors, reliable roofs, and lacking HWFs. In this way, they partly agree with JMP’s view on the risks associated with the use of certain types of latrines categorised as ‘unimproved’. The difference between JMP’s definition of ‘improved latrines’ and the general public’s viewpoint arises when the latter group tends to believe that if the latrine is well-constructed, easy to maintain, easy to clean, and the arrangement for regular cleaning after use, then it is not realistic to categorise it as ‘unimproved’ simply because it is being shared. We argue relying on this study’s evidence to support other authors who call for a review of WHO/UNICEF JMP’s definition for categorising latrines as ‘improved’ or ‘unimproved’. There is need for the debate to reach to an end if we come up with a better and commonly agreed definition. The national/country specific programme authorities strive to enhance community understanding of issues relating to sanitation and hygiene and participating in strategies aimed at scaling up the translation of JMP’s definition into practice at household level and to acknowledge the local contextual barriers, including the local interpretation of the dimensions of improved sanitation facilities. They have to use evidence from studies such as this one to review their interpretations and then package the promotional message using the languages and methodologies that can help to transform social understanding into better sanitation and hygiene behaviours. This is achievable because already there are programme-designed educational frameworks and messages that can be worked upon through review to integrate research evidence into workable definitions and acceptable sanitation behaviours in specific local contexts. Indeed, through the present study, we have been able to demonstrate a number of issues that are of significance to the promotion of sanitation and hygiene in LICs. These include how a combination of evidence garnered through researcher’s field observations and self-reports of the study populations provides an empirical basis for coming up with the indicators/inferences on what community members perceive on sanitation and hygiene issues. The study has also revealed how policy decision-makers and programme managers informed through this kind of research can appreciate the need for introducing necessary measures which are potential in enhancing or scaling-up of whatever seems to be novel and cost-effective intervention for supporting the attainment of the SDG of cutting-down the water, sanitation and hygiene related problems in LIC contexts.

## Supporting information

S1 File Data set in English language. (XLS)

S2 File Data set in Kiswahili language. (XLS)
